# Validation of a dynamic evaporation technique for generating isoflurane/air gas mixtures standards to calibrate anesthetic gas monitors

**DOI:** 10.1038/s41598-026-69018-z

**Published:** 2026-09-09

**Authors:** Aksam Abdelkhalik, Ghada Makhlouf, Randa N. Yamani

**Affiliations:** 1 Gas Analysis and Fire Safety Laboratory, National Institute of Standards (NIS), El‑Sadat Street, El‑Haram, P.O. Box 136, El‑Giza, 12211 Egypt; 2 Inorganic Analysis and Electrochemical Laboratory, National Institute of Standards (NIS), El‑Sadat Street, El‑Haram, P.O. Box 136, El‑Giza, 12211 Egypt

**Keywords:** Isoflurane–air mixtures, EN 1839, Dynamic evaporation, Calibration gas mixture, Anesthetic gas monitor, Method validation, Measurement uncertainty, Chemistry, Engineering, Medical research, Physics

## Abstract

The dynamic evaporation and mixing technique standardized in EN 1839 (method T), originally developed to determine the flammability limits of chemical vapors, was applied to prepare isoflurane-in-air calibration gas mixtures, with air serving as the carrier gas. Isoflurane concentrations spanning 0 to 8 vol% were generated on demand by independently controlling the liquid isoflurane and air flow rates. Verification using an anesthetic gas monitor demonstrated excellent agreement between predicted and measured concentrations. A comprehensive validation study was conducted in accordance with EURACHEM guidelines, encompassing linearity, limit of detection (LOD), limit of quantification (LOQ), precision, robustness, and measurement uncertainty. The method yielded a correlation coefficient of r² = 0.9998 over the full clinical range, an LOD of 0.08 vol%, and an LOQ of 0.27 vol%. All concentrations between 0.70 and 8 vol% complied with EN 1839 uncertainty requirements, confirming the suitability of this approach for routine calibration of isoflurane gas monitors.

**Supplementary Information:**

The online version contains supplementary material available at https://doi.org/10.1038/s41598-026-69018-z.

## Introduction

Accurate calibration of anesthetic gas monitors is essential for patient safety in clinical environments where volatile agents such as isoflurane are administered by inhalation. Current practice requires calibration using reference gas mixtures of known composition prepared in pressurized cylinders^1–8^. However, such cylinders present significant logistical, economic, and safety challenges: they are expensive to fill, difficult to transport, subject to strict storage requirements, and often unavailable in many countries, particularly in developing regions^1–5^. These limitations can compromise the traceability and frequency of monitor calibration, with potential consequences for patient safety.

Dynamic evaporation methods provide a practical on-demand alternative. By vaporizing a pure liquid agent directly into a controlled carrier-gas stream, these systems generate calibration mixtures continuously over a wide concentration range without the volume constraints inherent to pressurized cylinders.^8–13^ Critical parameters, including temperature, pressure, and flow rate, can be precisely controlled to achieve target concentrations, and the system can be reconfigured for different agents without specialized infrastructure^9–12^.

The European Standard EN 1839 (method T) utilizes a well-characterized dynamic evaporation apparatus, comprising a volumetric metering pump, a mass flow controller, and a temperature-controlled evaporator, to prepare precise vapor–oxidizer mixtures for explosion limit determination^14–17^. The procedure of EN 1839 (method T) can be applied to liquid vaporization into a controlled carrier gas stream. Consequently, the methodology can readily be extended to volatile inhalation anesthetics—including sevoflurane, enflurane, desflurane, and halothane—provided the following agent-specific adjustments are made: (1) the calculation model must incorporate the agent-specific molar mass (*M*), liquid density (*ρ*), and temperature-dependent vapor pressure (*P*_sat_); (2) agents with higher boiling points or lower vapor pressures (e.g., enflurane: b.p. 56.5 °C; sevoflurane: b.p. 58.6 °C) require precise temperature control at the evaporation chamber to prevent condensation or incomplete vaporization along the delivery line. Conversely, for highly volatile agents like desflurane (b.p. 22.8 °C), temperature regulation is critical to prevent uncontrolled boiling within the volumetric pump; and (3) seals, tubing, and volumetric pump materials must be verified for chemical inertness against each specific liquid anesthetic to avoid leaching or sorption losses. The practical applicability and validation of this dynamic evaporation framework across different anesthetic agents (sevoflurane in air and oxygen, and isoflurane in oxygen) have been successfully demonstrated in our previous work. The results indicated that the measured concentrations showed very good agreement with the target concentrations, and the measurement uncertainty was within the acceptable limits specified by the standard method EN 1839^8,14^.

Isoflurane has high vapor pressure (330 mmHg at 25 °C), making it highly suitable for dynamic evaporation systems^18^. Despite its widespread clinical use^8^, no published study has specifically validated a dynamic preparation method for isoflurane-in-air calibration gas mixtures against a comprehensive set of analytical performance criteria. The EN 1839 (method T) apparatus, therefore, presents an opportunity to fill this gap.

The objective of this study was therefore to apply and fully validate the EN 1839 method T apparatus for the preparation of isoflurane-in-air calibration gas mixtures, in accordance with EURACHEM method validation guidelines^19^. The validation includes GUM-based uncertainty evaluation and inter-instrument comparison across multiple optical interferometric anesthetic gas monitors, to establish EN 1839 as a practical, cylinder-free calibration platform for volatile anesthetic agents under controlled laboratory conditions^19^.

## Experimental setup

### Materials

The carrier gas—compressed air containing O_2_ (20.95%), N_2_ (78.08%), CO_2_ (0.03%), and Ar (0.93%)—used in the experiments was Instrument-Grade Clean Dry Air (CDA) generated via an oil-filtered laboratory air compressor. To ensure the air was suitable for precise dynamic evaporation and calibration mixture preparation, the compressed air stream passed through an oil-removal filter to eliminate trace compressor oil aerosols and hydrocarbon vapors. The air was continuously dried using an inline silica gel desiccant column to strip residual moisture. As an additional safeguard prior to sensor detection, an inline anhydrous calcium chloride (CaCl_2_) filter (for moisture removal) was positioned directly before the anesthetic gas monitor’s inlet.

Pure isoflurane (100% purity, CAS 26675-46-7, MW 184.49 g/mol, liquid density 1.496 g/mL at 20 °C, vapor pressure 330 mmHg at 25 °C) was produced by El-Amria for Pharmaceutical Industries (for Pharco Pharmaceuticals), Egypt, and it was used as received.

### EN 1839 (method T) instrument and mixture preparation

**Fig. 1:**
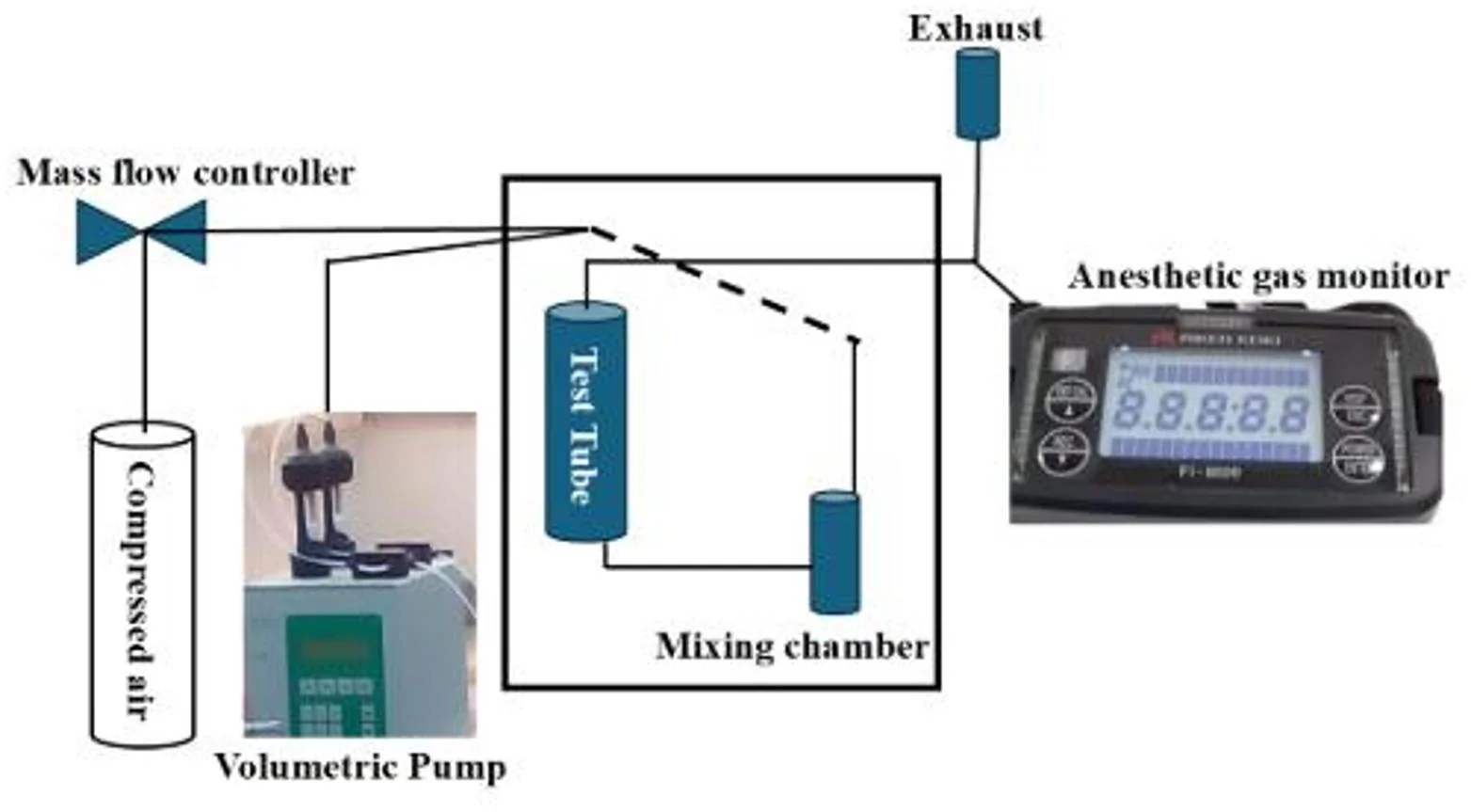
Schematic representation for EN 1839 method T instrument used in preparation of isoflurane in air mixtures to calibrate anesthetic gas monitors.

Isoflurane–air mixtures were prepared following EN 1839 (method T),^8,14–17^ as illustrated in Fig. 1. The apparatus consists of a volumetric metering pump, a compressed-air source regulated by a calibrated mass flow controller, a glass evaporator, and a 600 mL glass mixing chamber. The evaporator was maintained at 37 ± 1 °C within a temperature-controlled heating chamber, inclined at 60°. Liquid isoflurane was delivered onto the inner-glass wall of the evaporator via a stainless-steel needle. The vapor–air mixture was homogenized in the 600 mL chamber before measurement. All operations were performed under a continuous-extraction fume hood. Before collecting data, the system was purged with ten times the volume of the test tube. The total flow rate was maintained at 2 L/min unless otherwise stated. The calculation model and an example illustrating its application are presented in the Supplementary Data file (part A).

### Measurement

Isoflurane concentrations were measured using a RIKEN Anesthetic Gas Monitor (Model F1-8000, RIKEN KEIKI Co., Ltd., Japan), based on the optical interferometric principle: the gas concentration is derived from the shift in interference fringes proportional to the refractive index difference between the sample and reference air cells^8,20^. Measuring range: 0–8 vol%, resolution: 0.01 vol%. Predicted concentrations were calculated using the ideal gas law, considering liquid flow rate, density, molecular weight, and total flow rate. Measurements were performed at 25 °C ± 0.4 °C and RH = 46.5 ± 1.7%. Each concentration represents the arithmetic mean of six independent replicates (*n* = 6).

### Method validation

Validation was conducted in accordance with EURACHEM fitness-for-purpose guidelines, covering linearity, limit of detection (LOD), Limit of quantification (LOQ), trueness/bias, precision (intraday, interday, intersystem), robustness, and measurement uncertainty^19^.

#### Linearity

The linear relationship between predicted (Y-axis) and mean measured (X-axis) concentrations was evaluated across seven nominal levels spanning 0–8 vol% (*n* = 6 per level; *N* = 42) using ordinary least-squares (OLS) linear regression. Variance homogeneity across concentration levels was assessed using Bartlett’s test^21,22^.

#### Trueness (bias)

Trueness was assessed by comparing predicted concentrations (serving as reference values) with mean measured concentrations at each level. Absolute bias (b = measured − predicted) and relative bias (b%) were calculated. Recovery (%) was computed as (measured/predicted) × 100^19, 23^. The statistical significance of the mean bias was assessed using a one-sample t-test against zero^23^.

#### Selectivity

The potential impact of interferents on the RIKEN F1-21 optical interferometric measurement was evaluated through a theoretical analysis of the measurement principles and an examination of published cross-sensitivity data about comparable monitoring systems^2,24–26^.

#### LOD and LOQ

The minimum achievable concentration (0.15 vol%) was measured ten times (*n* = 10). LOD and LOQ were computed as 3 × S₀* and 10 × S₀*, respectively. S₀ represents the standard deviation of the *n* = 10 replicate measurements performed at the minimum achievable concentration, used directly for the calculation of detection and quantification limits, in accordance with EURACHEM guidelines^23^.

#### Precision

The precision was evaluated at 1, 3, and 5 vol% across three tiers: (i) intraday repeatability (*n* = 6, single session); (ii) interday intermediate precision (*n* = 6, different day); and (iii) intersystem reproducibility (*n* = 6, second independently calibrated RIKEN F1-21). Results are presented as relative standard deviation (RSD) in percentage.

#### Robustness

The effect of total flow rate variation (1.0, 1.5, 2.0 L/min) on the measured concentration at 3 vol% was assessed (*n* = 6 per condition).

#### Measurement uncertainty

The expanded uncertainty (U) was computed from nine identified uncertainty sources combined in quadrature, multiplied by k = 2 (~ 95% confidence), following the GUM-based EURACHEM/CITAC approach^27–31^.

### Inter-instrument verification

To assess the reliability and transferability of the validated dynamic isoflurane–air preparation method across various measurement units, four optical interferometric anesthetic gas monitors of the same model (RIKEN F1-21, RIKEN KEIKI Co., Ltd., Japan) were utilized to independently measure calibration gas mixtures prepared at target concentrations of 0, 1, 3, and 5 vol%. Each instrument was operated under identical conditions, utilizing the same EN 1839 method T apparatus, with a controlled ambient temperature of 25 ± 0.4 °C, relative humidity of 46.5 ± 1.7%, and a total flow rate of 2 L/min. Before measurement, each unit was independently zeroed against clean compressed air. Six replicate readings were recorded for each instrument at each concentration level (*n* = 6 per instrument per level), resulting in a total of 96 measurements across all four units.

## Results and discussion

### Accuracy and predicted vs. measured concentrations

Table 1 presents a summary of measurement accuracy across the full clinical range of 0 to 8 vol%. The absolute error, calculated as the difference between the measured and predicted values, ranged from − 0.07 to + 0.05 vol%. Notably, this error remained at or below the monitor’s inherent accuracy specification for all concentrations starting from 1 vol% and above. The monitor accuracy as specified by the manufacturer is given by Eq. (1):^20^

1 $$\:Accuracy=\pm\:(reading - \alpha)\times\:0.03\pm \beta$$

where α is the air-calibration offset (0 vol%) and β is a drift term (0 vol% under the stable ambient conditions of this study: 25 °C ± 0.4 °C; RH = 46.5 ± 1.7%). In our measurements, the air calibration value was recorded as zero vol%. The symbol (β) denotes the drift in indication that may occur due to ambient temperature fluctuations of ± 10 ℃.^20^ Since our measurements were conducted at a temperature of 25 ± 0.4 ℃ and relative humidity of 46.5 ± 1.7%, they fell within the operating conditions specified for the anesthetic gas monitor (5–35 ℃ and RH < 80%). Consequently, the β value was also zero vol%.

### Trueness (bias) and recovery

Trueness was evaluated by calculating the absolute bias, relative bias, and recovery (%) at each concentration level, as summarized in Table 1. Trueness was assessed by calculating the absolute bias at each concentration level as the difference between the mean measured and the predicted concentration. The mean absolute bias across the six non-zero concentration levels was − 0.007 vol%, indicating negligible systematic error. Recovery values ranged from 98.7% to 126.7%, with the anomalous recovery at 0.15 vol% (126.7%) that may be attributed to the integer-only programming limitation of the volumetric pump at the minimum flow rate, rather than a systematic method error and it was excluded from the calculation of the mean recovery for the clinically relevant range (0.70–8 vol%) but retained in the overall bias summary and t-test as a conservative assessment. Excluding this level, the mean recovery across the clinically relevant range (0.70–8 vol%) was 101.1%, well within the 95–105% acceptance range recommended by EURACHEM^19,23^.

**Table 1:** Trueness assessment: absolute bias, relative bias, and recovery at each concentration level of isoflurane concentrations in air.

Predicted (vol%)	Measured (vol%)	Absolute bias (vol%)	Relative bias (%)	Recovery (%)
0.15	0.19	+ 0.04	+ 26.7	126.7
0.70	0.75	+ 0.05	+ 7.1	107.1
1.00	1.01	+ 0.01	+ 1.0	101.0
3.00	2.96	−0.04	−1.3	98.7
5.00	4.97	−0.03	−0.6	99.4
8.00	7.93	−0.07	−0.9	99.1
Mean (all levels)		**−0.007**	**+ 5.3%**	**105.3%**
Mean (0.70–8 vol%)		−0.016	+ 1.1%	101.1%
t-test (bias vs. 0)		t = − 0.343	*p* = 0.746	Not significant

To determine whether the observed bias was statistically significant, a two-sided one-sample t-test was applied to the six bias values against a reference value of zero, using Eq. (2):

2 $$\:t=\frac{\stackrel{-}{b}}{S/\sqrt{n}}$$

where $\:\stackrel{\prime }{b}\:$is the mean bias (vol%), * S* is the standard deviation of the bias values, and * n* is the number of concentration levels evaluated. The test results that, $\:t=-0.343\:$(df = * n-1*= 5, * p*= 0.746), which is less than the critical value $\:{t}_{critical}$= 2.571 at $\:\alpha\:\:$= 0.05 (two-tailed). Since $\:\mid\:{t}_{calc}\mid\:$< $\:{t}_{critical}$, the null hypothesis (no systematic bias) was not rejected, confirming that the mean bias is not statistically significant at the 5% significance level (* p* > 0.05). The method demonstrates satisfactory accuracy within the validated concentration range^19^.

### Selectivity

Selectivity refers to the ability of an analytical method to measure the target analyte accurately in the presence of other components that may be present in the sample matrix and may interfere with the target analyte. The RIKEN F1-21 anesthetic gas monitor operates on the optical interferometric principle, in which the concentration of the target gas is determined from the difference in refractive index between the sample gas stream and a reference air cell. Since this measurement is based on bulk physical property rather than a chemical-specific signal, the instrument will respond to any gas whose refractive index differs from that of air. In the present study, the gas mixtures consisted mainly of pure isoflurane vapor in air, with no other volatile compounds present in the system. Selectivity was therefore ensured by experimental design: the compressed air carrier gas was free of volatile organic compounds, and the system was zeroed against the uncontaminated air stream before each measurement sequence, effectively compensating for any background refractive index contribution. Under these controlled laboratory conditions, cross-interference from other compounds was not a concern. It is essential to recognize that the optical interferometric principle presents a limitation in selectivity during clinical or field deployment. Published studies on anesthetic gas monitors have documented cross-sensitivity to several compounds commonly present in operating-room environments, including nitrous oxide (N₂O), carbon dioxide (CO₂) at clinical concentrations (~ 5 vol%), isopropanol and ethanol vapors from disinfectants, water vapor, and other volatile anesthetic agents such as sevoflurane and desflurane if present simultaneously^25,26^. These interferents can produce false-positive readings or bias the measured isoflurane concentration. The method validated in the present study is therefore specifically applicable to controlled laboratory calibration environments where the gas stream composition is known and controlled. For clinical in-situ applications, it is recommended to verify the absence of significant interferents or to apply appropriate background corrections consistent with the monitor manufacturer’s guidance. A formal experimental selectivity study using potential interferents at clinically relevant concentrations is recommended as a direction for future work.

### Linearity

Linear regression analysis of 42 data points at seven concentration levels yielded Eq. (3).

3 $$\:Y\hspace{0.17em}=\hspace{0.17em}1.0101X\hspace{0.17em}-\hspace{0.17em}0.0155$$

**Fig. 2:**
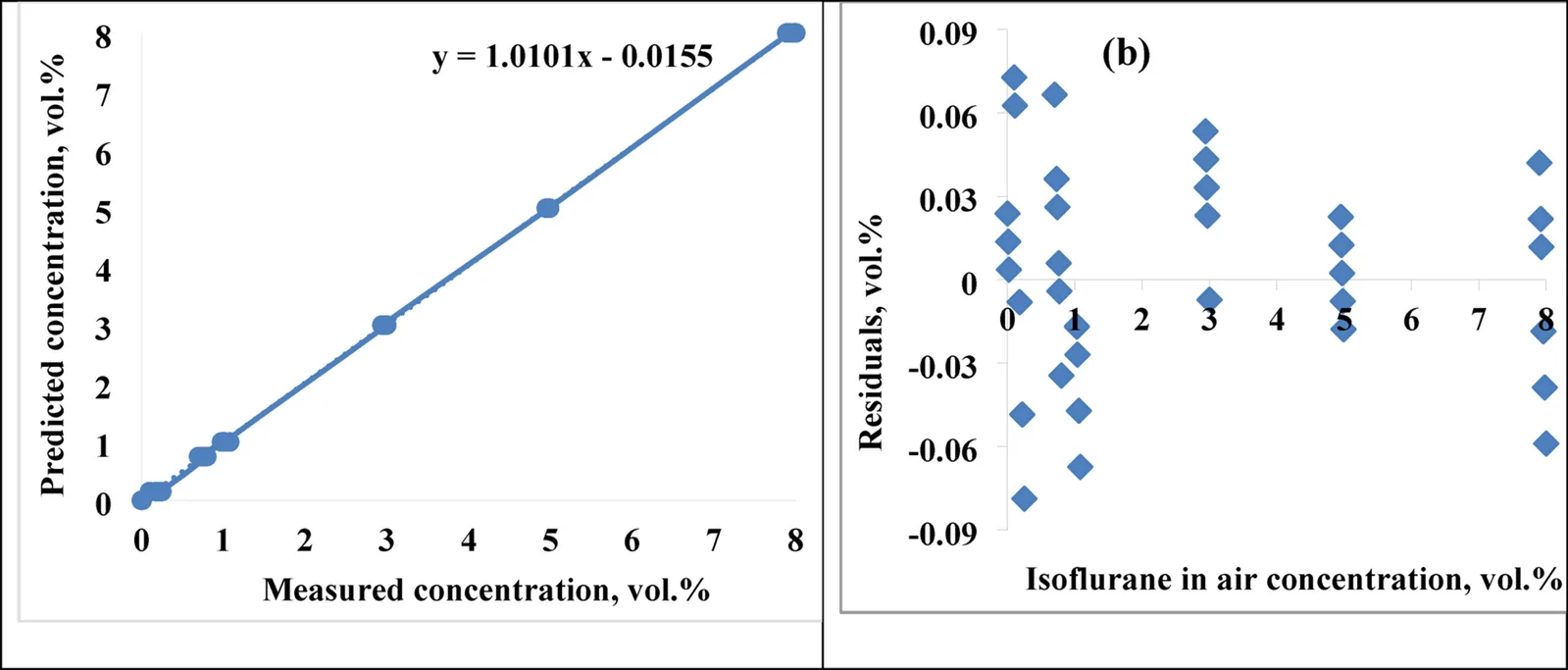
(**a**) Relationship between predicted and mean measured isoflurane concentrations; (**b**) residuals plot.

Where r² = 0.9998 as shown in Fig. 2a. The slope of 1.0101, deviating by only 1.01% from unity, confirms negligible proportional bias, while the near-zero intercept (0.0155 vol%) indicates minimal constant systematic offset. The residual plot (Fig. 2b) shows random, zero-centered residuals with no systematic pattern.

Bartlett’s test for variance homogeneity across the seven concentration levels yielded χ² = 26.354 (df = 6, *p* < 0.001), indicating statistically significant heteroscedasticity: the measurement variance increases with concentration, as expected for a gas-phase volumetric system where absolute uncertainty scales proportionally with signal level^21,22,28^. While OLS regression remains a valid and widely used approach under these conditions, given the small absolute magnitude of the deviations (maximum STDEV = 0.04 vol% at all levels).

To account for the heteroscedasticity revealed by Bartlett’s test, a weighted linear regression was additionally performed using weights (w_i_) inversely proportional to the measurement variance (s_i_) at each concentration level (w_i_ = 1/s_i_²)^21,22,28^. The resulting weighted calibration line was presented by Eq. (4), with a weighted coefficient of determination $\:{R}_{w}^{2}=0.99999$, very close to the ordinary least-squares line obtained from the unweighted data.

4 $$\:{\mathrm{Y}}_{\mathrm{w}}=0.0067+0.9917\mathrm{X}\:$$

The small difference between the two regression lines over the 0–8 vol% range indicates that, although the measurement variances are statistically heterogeneous, this heteroscedasticity has a negligible practical impact on the assigned concentrations for calibrating isoflurane gas monitors^8,19,29^.

### LOD and LOQ

At the minimum achievable concentration (0.15 vol%), ten replicate measurements yielded an LOD of 0.08 vol% and an LOQ of 0.27 vol% using the EURACHEM approach $\:LOD=3{\:\times\:S}_{0}$, $\:LOQ=10\times\:{S}_{0}$, where $\:{S}_{0}$ is the standard deviation at the minimum level. The LOD of 0.08 vol% indicates that the system can generate and detect isoflurane at concentrations well below the standard therapeutic range (1.0–3.0 vol%), supporting sensitive calibration at low clinical settings. The LOQ of 0.27 vol% defines the lower limit for precise quantitative measurement in this dynamic preparation system and confirms its suitability for routine calibration of anesthetic gas monitors over the 0.70–8 vol% range^19^. The LOQ/LOD ratio of approximately 3.4 is consistent with the expected signal-to-noise relationship for gas-phase optical interferometric detection. Together with the high linearity (r² = 0.9998), these values demonstrate that baseline noise is stable and that the regression model provides a reliable framework for assigning calibration concentrations.

### Precision

Precision results are summarized in Table 2. Intraday RSDs of 3.80%, 1.01%, and 0.60% at 1, 3, and 5 vol%, respectively, confirm excellent within-session repeatability. The inverse concentration–RSD relationship reflects the proportionally higher absolute signal relative to baseline noise at higher concentrations. Interday RSDs (3.88%, 1.00%, 0.62%) and intersystem RSDs (2.80%, 1.03%, 0.40%) confirm transferability across time and instrumentation. All RSD values remain well below the 5% threshold accepted for gas-mixture analytical methods^19,24^.

**Table 2:** Intraday, interday, and intersystem precision data of isoflurane in air.

Parameter	Predicted concentration, vol%	Mean of measured concentration, vol%	STDEV, vol%	RSD, %
Intraday	1.00	1.05	0.04	3.80
	3.00	2.96	0.03	1.01
	5.00	4.99	0.03	0.60
Interday	1.00	1.03	0.04	3.88
	3.00	3.00	0.03	1.00
	5.00	4.91	0.03	0.62
Intersystem	1.00	1.06	0.03	2.80
	3.00	3.01	0.04	1.03
	5.00	5.00	0.02	0.40

### Robustness

Total flow rates were deliberately varied from 1.0 to 2.0 L/min at a fixed target concentration of 3.0 vol%, yielding measured concentrations of 2.96, 2.98, and 3.01 vol% with a maximum absolute deviation of 0.05 vol% (Table 3). The calculated air and vapor flow rates required to achieve a concentration of 3.0 vol% at total flow rates of 1.0, 1.5, and 2.0 L/min are presented in the Supplementary Data file (part B). The relative standard deviation (%RSD) ranged from 1.00% to 1.32%, well within the acceptable upper limit of $\:\le\:5.0\%$ (and meeting the stringent target of $\:\le\:2.0\%$ for major component analysis) defined in analytical validation guidelines^19^. These findings confirm that the proposed dynamic evaporation system is robust against operational flow rate fluctuations, such as those caused by mass flow controller drift.

**Table 3:** The data for method robustness.

Mixture composition	Total flow rate (L/min)	Predicted concentration, vol%	Measured concentration, vol%	STDEV, vol%	RSD, %
Isoflurane in air	1.00	3.00	2.96	0.03	1.01
	1.50	3.00	2.98	0.03	1.00
	2.00	3.00	3.01	0.04	1.32

### Measurement uncertainty

Measurement uncertainty was evaluated using a bottom-up approach in accordance with ISO/IEC Guide 98 − 3 (GUM) and EURACHEM/CITAC guidance^27–31^. The measurand is the isoflurane concentration in air (* C*(vol%)), generated by the EN 1839 method T apparatus and measured with the anesthetic gas monitor. The uncertainty model consists of a Type A component (repeatability of the monitor readings) and several Type B components associated with the main physical and instrumental factors of the dynamic preparation process: gas monitor accuracy, evaporator and ambient temperatures, mass flow controller calibration, pump flow rate, relative humidity, and isoflurane purity. For each concentration level, the Type A standard uncertainty $\:{u}_{A}$was obtained from the standard deviation of six replicate readings divided by $\:\sqrt{n}$, (*n* = 6). Type B standard uncertainties were derived from manufacturer specifications, calibration data, or reasonable assumptions, and converted into concentration units using appropriate sensitivity coefficients. Gas monitor accuracy was treated as a rectangular distribution, with the standard uncertainty equal to the specified accuracy divided by $\:\sqrt{3}$. Temperature effects (evaporator and ambient) were quantified by assessing the influence of the corresponding temperature range (± 0.4 °C) on the vaporization and refractive index and expressed as standard uncertainties in vol%. The mass flow controller and pump flow uncertainties were evaluated as relative contributions to the prepared concentration, while relative humidity and purity were treated as small constant or proportional terms. All contributions were combined by root-sum-of-squares to obtain the combined standard uncertainty $\:{u}_{c}$, and the expanded uncertainty * U*was calculated as $\:U=k{\hspace{0.17em}}{u}_{c}$with a coverage factor $\:k=2\:$(approximately 95% confidence). Table 4 summarizes the individual Type A and Type B sources, their values, probability distributions, and divisors, whereas Table 5 presents the complete uncertainty budget at each concentration level.

**Table 4:** Analysis of uncertainty components.

Uncertainty type	Uncertainty component	Uncertainty value (u)	Divisor	Distribution
Type A	Repeatability of measurement	$\:\frac{STDEV}{\sqrt{n}}$	$\:\sqrt{6}$	Normal
Type B	Gas monitor accuracy	(0.03 × instrument reading) vol%	$\:\sqrt{3}$	Rectangular
	Pump	0.01 L/h	1	Normal
	Mass flow controller	2 mL/min	1	Normal
	Evaporator temperature	0.4 ℃	1	Normal
	Purity of isoflurane	0.05%	$\:\sqrt{3}$	Rectangular
	Ambient temperature	0.4 ℃	1	Normal
	Relative humidity	1.7%	1	Normal
	Stopwatch	0.094 s/day	1	Normal

As an illustrative example, the uncertainty calculation at 3 vol% is outlined here. Six replicate readings at 3 vol% yielded a Type A standard uncertainty of $\:{u}_{A}=0.0084\:$vol%. The gas monitor’s accuracy at this level gives $\:u\left(\mathrm{monitor}\right)=0.0519\:$ vol% assuming a rectangular distribution, while the contributions from the evaporator and ambient temperature were each $\:0.0038\:$vol%. The remaining Type B contributions were $\:u\left(\mathrm{MFC}\right)=0.0060$ vol%, $\:u\left(\mathrm{pump}\right)=0.0165$ vol%, $\:u\left(\mathrm{RH}\right)=0.001$ vol%, $\:u\left(\mathrm{purity}\right)=0.00001$ vol%, and $\:u\left(\mathrm{stopwatch}\right)\approx\:0.000003\:$vol%. The combined standard uncertainty was therefore calculated by the root-sum-of-squares of all Type A and Type B terms, using the following equation:

$$\:{u}_{c}=\:\sqrt{{u}_{A\:\:}^{2}+{u\left(Monitor\right)}^{2}+{u\left(Evaporator\:temp\right)}^{2}+{u\left(Pump\right)}^{2}+{u\left(stopwatch\right)}^{2}+{u\left(MFC\right)}^{2}}$$

to give $\:{u}_{c}\approx\:0.055\:$vol%, and the expanded uncertainty was $\:{U}_{\mathrm{e}\mathrm{x}\mathrm{p}}=2{u}_{c}\approx\:0.11\:$vol%. corresponding to a relatively expanded uncertainty of approximately 4.8% at 3.00 vol%. Similar calculations were performed at all concentration levels. The resulting expanded uncertainties (U, k = 2) ranged from about ± 0.004 vol% at 0 vol% to ± 0.20 vol% at 8 vol%, with relative expanded uncertainties decreasing from 42.8% at 0.15 vol% (below the LOQ) to around 6.8–2.6% over the 0.70–8 vol% range.

**Table 5:** GUM-based uncertainty budget for isoflurane-in-air calibration gas mixtures (k = 2, ~ 95% confidence.

Predicted (vol%)	Mean measured (vol%)	$\:{\boldsymbol{u}}_{\boldsymbol{c}}$	$\:\boldsymbol{U}$(vol%)
0.00	0.007	0.002	0.004
0.15	0.18	0.03	0.06
0.70	0.75	0.02	0.05
1.00	1.01	0.03	0.06
3.00	2.96	0.05	0.11
5.00	4.97	0.08	0.17
8.00	7.93	0.10	0.20

When evaluated against the EN 1839 accuracy requirements, ± 10% relative for molar amounts of test substance ≤ 2% and ± 0.2% absolute for molar amounts > 2%,^18^ the prepared isoflurane–air mixtures performed well. All concentrations between 0.70 and 8 vol% complied with these criteria, whereas the lowest level (0.15 vol%) exceeded the allowable uncertainty threshold due to pump programming limitations and its position below the LOQ. As a consistency check, a top-down uncertainty estimate based on method performance data (repeatability, intermediate precision, inter-system reproducibility, and bias) at 1, 3, and 5 vol% yielded expanded uncertainties of approximately 0.064–0.071 vol%, which are slightly smaller than the GUM-based values and confirm that the bottom-up uncertainty budget adopted here is conservative.

### Inter-instrument comparison

The anesthetic gas monitor (RIKEN FI-21) operating principle is based on optical refractometry (interferometry), where gas concentration is calculated relative to a reference gas constant. Prior to each measurement series, the four RIKEN FI-21 monitors underwent zero-point baseline calibration (AIR CAL) using purified, dry compressed air, following the manufacturer’s technical guidelines. In the absence of commercially available certified reference gas cylinders (CRMs) for volatile anesthetic agents in Egypt, traceability was established primary-way using the dynamic evaporation system. All critical physical parameter devices governing the generated gas concentration were individually calibrated with SI traceability at the National Institute of Standards (NIS), Egypt. The volumetric pump delivering liquid anesthetic agent was calibrated gravimetrically using calibrated analytical balances traceable to SI mass standards. Mass Flow Controller (MFC), for controlling the flow rate of air, was calibrated using NIS standard flowmeters. Digital timer was calibrated against national frequency/time standards. Thermocouples and relative humidity sensors were calibrated against NIS working reference standards. The dynamically generated concentration was consequently assigned from a traceable measurement model and used to assess monitor response and linearity. The calculated concentration of the dynamic gas mixture generated by the system served as the primary reference standard against which the response and linearity of the four RIKEN FI-21 monitors were studied.

**Fig. 3:**
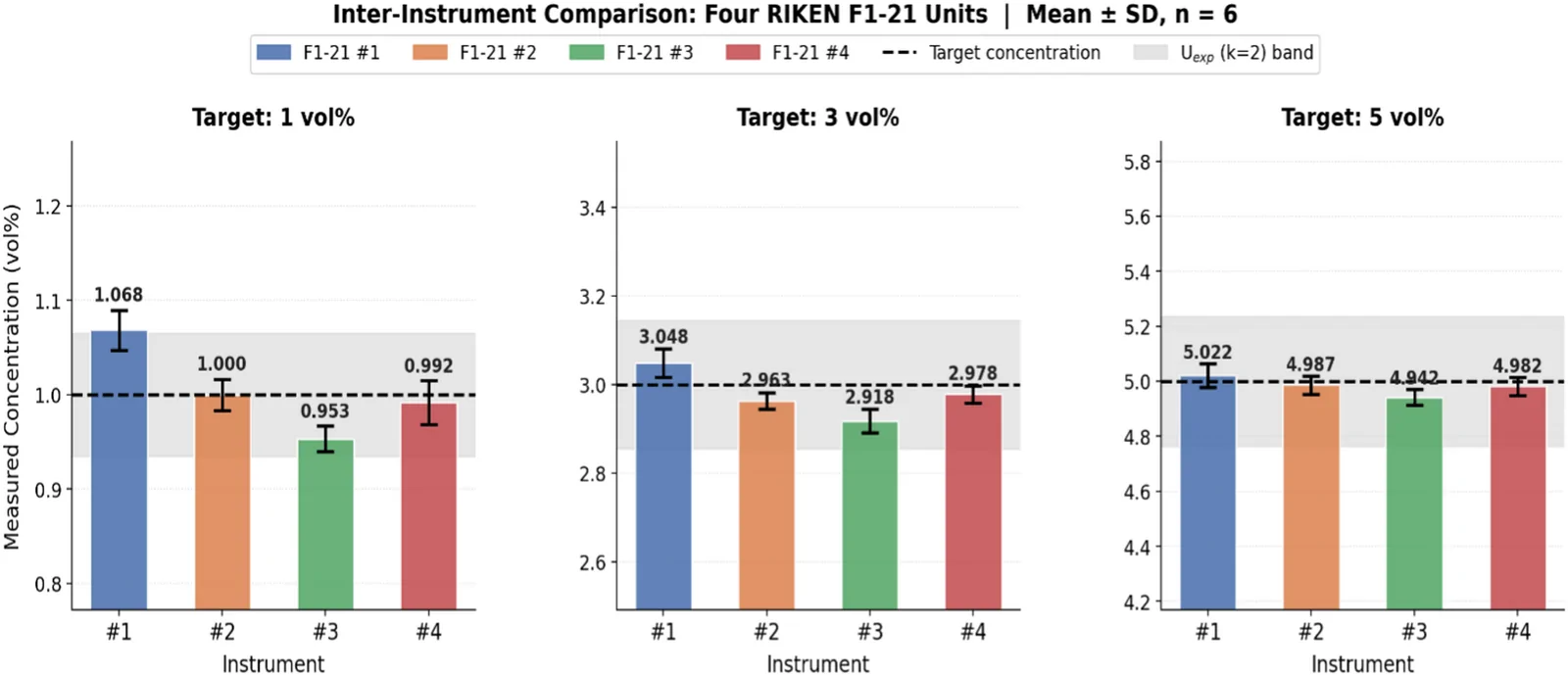
Inter-instrument comparison of four optical interferometric anesthetic gas monitors using prepared isoflurane–air calibration mixtures at target concentrations of 1, 3, and 5 vol%.

The measured concentrations of all four instruments are presented in Fig. 3 as mean ± SD (*n* = 6 per instrument per concentration level), together with the target concentration (horizontal bar) and the expanded uncertainty of the validated preparation method (U), k = 2, shown as the grey band. At three concentration levels (1, 3, and 5 vol%), the mean values of all four instruments fall within the expanded uncertainty band, confirming that the dynamic EN 1839 preparation method produces calibration mixtures that are consistent and fit for purposes across multiple measurement units.

## Conclusions

This study validates a robust, on-demand dynamic evaporation methodology based on EN 1839 for the calibration and validation of anesthetic gas monitors. Compared to traditional static cylinder-based gas standards, this dynamic approach offers significant novel advantages: it eliminates long-term storage instability and surface-adsorption losses of volatile halogenated agents, provides direct SI metrological traceability via primary physical mass/flow parameters, and allows continuous, multi-point concentration generation across wide clinical ranges. By offering a cost-effective, highly accurate, and standardized alternative to commercial reference cylinders, this methodology provides metrology institutes and clinical calibration laboratories with a reliable framework for routine quality assurance of anesthetic monitors. The method showed excellent analytical performance over the validated concentration range, with outstanding linearity (r² = 0.9998), satisfactory sensitivity (LOD = 0.08 vol%, LOQ = 0.27 vol%), high precision (RSD ≤ 3.88%), and no statistically significant overall bias. For the clinically relevant quantitative range of 0.70–8 vol%, the mean recovery was 101.1%, and all concentrations complied with the EN 1839 accuracy and uncertainty requirements. The method also proved robust to moderate variation in total flow rate, supporting its practical use for routine preparation of calibration mixtures. In addition, inter-instrument verification at 1, 3, and 5 vol% demonstrated good agreement among independently calibrated optical interferometric gas monitors, indicating that the prepared mixtures are reproducible across measurement units within the tested range. These findings support the applicability of the EN 1839 dynamic evaporation approach as a practical calibration platform for anesthetic gas monitoring, particularly in settings where certified pressurized gas cylinders are expensive, difficult to transport, or not readily available.

## Supplementary Information

Below is the link to the electronic supplementary material.

Supplementary Material 1

## Data Availability

Processed data are presented within the manuscript. Raw data are available on reasonable requests from the corresponding author.
