# Supplementary material for: Validation of a dynamic evaporation technique for generating isoflurane/air gas mixtures standards to calibrate anesthetic gas monitors
Source: Sci Rep. 2026 Sep 9;16:28220. doi: 10.1038/s41598-026-69018-z (PMC13558684; doi:10.1038/s41598-026-69018-z)
Supplement: Supplementary file 1 [file 41598_2026_69018_MOESM1_ESM.docx]

**Validation of a Dynamic Evaporation Technique for Generating Isoflurane/Air Gas Mixtures Standards to Calibrate Anesthetic Gas Monitors**

Aksam Abdelkhalik¹^,^*, Ghada Makhlouf¹, Randa N. Yamani²

¹ Gas Analysis and Fire Safety Laboratory, National Institute of Standards (NIS), El‑Sadat Street, El‑Haram, P.O. Box 136, El‑Giza 12211, Egypt
² Inorganic Analysis and Electrochemical Laboratory, National Institute of Standards (NIS), El‑Sadat Street, El‑Haram, P.O. Box 136, El‑Giza 12211, Egypt

*Corresponding author: Aksam Abdelkhalik
Corresponding author: [aksam.abdelkhalik@nis.sci.eg](mailto:aksam.abdelkhalik@nis.sci.eg)

**Part A**

**1. Calculation Model**

The volumetric concentration ($C$, in vol.%) of the vaporized liquid anesthetic (isoflurane) in the dry carrier gas (air) stream is calculated using the ideal gas law relationship for dynamic evaporation:

$$C=\frac{V_{\text{vap}}}{V_{\text{vap}}+V_{\text{carrier}}}\times100\%$$

Expressed in terms of operational laboratory parameters (liquid delivery rate and gas flow rate):

$$C=\frac{\left( \frac{q_{l}\cdot\rho}{M} \right)\cdot V_{m}}{\left( \frac{q_{l}\cdot\rho}{M} \right)\cdot V_{m}+Q_{g}}\times100\%$$

**Where:**

$C$: Calculated volumetric concentration of isoflurane in the gas mixture (vol.%).

$q_{l}$: Liquid infusion rate of liquid isoflurane delivered by the pump (mL/min).

$\rho$: Density of liquid isoflurane at the evaporation temperature (g/mL, e.g., 1.496 g/mL at 20 ℃)

$M$: Molar mass of isoflurane (184.49 g/mol).

$V_{m}$: Molar volume of ideal gas at standard operating temperature and pressure ($22.414\text{ L/mol}$ at STP, or adjusted to ambient operating $T$ and $P$ via $V_{m}=\frac{R\cdot T}{P}$).

$Q_{g}$: Volumetric flow rate of the air measured by the calibrated MFC ($\text{L/min}$ or $\text{SLM}$).

$V_{\text{vap}}$: Vaporized volume rate of isoflurane ($\text{L/min}$).

**2. Operating Assumptions**

a) Complete evaporation: Complete $100\%$ phase change of the liquid anesthetic into vapor without thermal decomposition or partial condensation in the delivery line.
b) Ideal gas behavior: The vaporized anesthetic and carrier gas behave as ideal gases under working temperature ($T$) and pressure ($P$) conditions.
c) Steady-state delivery: Constant and non-pulsating liquid infusion rate ($q_{l}$) and carrier gas flow rate ($Q_{g}$).
d) Homogeneous mixing: Thorough and complete mixing of the vaporized agent with the air stream before entering the measurement chamber.

**3. Step-by-Step example using parameters for isoflurane:**

To calculate the theoretical volumetric concentration ($C$) of isoflurane generated under the following standard laboratory conditions at ${20}^{\circ}\text{C}$ **(**$293.15\text{ K}$**)** and $1\text{ atm}$ **(**$101.325\text{ kPa}$**)**:

**Experiment parameters:**

Liquid infusion rate ($q_{l}$): $0.15\text{ mL/min}$ (delivered by the volumetric pump)

Air flow rate ($Q_{g}$): $1.50\text{ L/min}$ (air flow controlled by the MFC)

Liquid isoflurane density ($\rho$): $1.496\text{ g/mL}$ (at ${20}^{\circ}\text{C}$)

Molar mass of isoflurane ($M$): $184.49\text{ g/mol}$

Molar volume of ideal gas ($V_{m}$): $24.055\text{ L/mol}$ at ${20}^{\circ}\text{C}$ and $1\text{ atm}$

*(Note:* $V_{m}=\frac{R\cdot T}{P}=\frac{0.08206\cdot293.15}{1}=24.055\text{ L/mol}$*)*

**Step-by-Step Calculation**

**Step 1: Calculate the molar flow rate of isoflurane (**$\boldsymbol{n}_{\text{iso}}$**)**

First, determine how many moles of liquid isoflurane are delivered per minute:

$$\text{Mass }\text{f}\text{low rate}=q_{l}\times\rho=0.15\text{ mL/min}\times1.496\text{ g/mL}=0.2244\text{ g/}\text{min}$$

$$n_{\text{iso}}=\frac{q_{l}\cdot\rho}{M}=\frac{0.2244\text{ g/}\text{min}}{184.49\text{ g/mol}}\approx0.0012163\text{ mol/min}$$

**Step 2: Calculate the vaporized gas volume rate (**$\boldsymbol{V}_{\text{vap}}$**)**

Convert the molar flow rate of liquid into the equivalent vapor volume generated per minute using the molar volume ($V_{m}$):

$$V_{\text{vap}}=n_{\text{iso}}\times V_{m}=0.0012163\text{ mol/min}\times24.055\text{ L/mol}\approx0.02926\text{ L/}\text{min}$$

*(So,* $0.15\text{ mL}$ *of liquid isoflurane yields approximately* $0.02926\text{ L}$ *or* $29.26\text{ mL}$ *of pure gas vapor per minute).*

**Step 3: Calculate total gas stream volume (**$\boldsymbol{V}_{\text{total}}$**)**

Combine the vaporized gas rate with the carrier gas flow rate ($Q_{g}=1.50\text{ L/min}$):

$$V_{\text{total}}=V_{\text{vap}}+Q_{g}=0.02926\text{ L/min}+1.50\text{ L/min}=1.52926\text{ L/min}$$

**Step 4: Calculate final concentration (**$\boldsymbol{C}$**)**

**In percent volume (vol%):**

$C=\frac{V_{\text{vap}}}{V_{\text{total}}}\times100\%=\frac{0.02926\text{ L/min}}{1.52926\text{ L/min}}\times100\%\approx1.913$vol.%

In parts per million ($\text{ppm}_{\text{v}}$):

$$C_{\text{ppm}}=\frac{V_{\text{vap}}}{V_{\text{total}}}\times{10}^{6}=\frac{0.02926}{1.52926}\times{10}^{6}\approx19,133\text{ ppm}_{\text{v}}$$

**Note:** it is necessary to consider corrections obtained from calibration certificates of instruments used such as mass flow controller and volumetric pump.

**Part B**

**Method robustness**

**Calculation of air flow rate and liquid flow rate to prepare concentration 3.0 vol.% where the total flow rate is 1.0, 1.5, and 2.0 L/min.**

When the total gas flow rate ($Q_{\text{total}}=V_{\text{vap}}+Q_{g}$) is fixed at specific setpoints ($1.0$, $1.5$, and $2.0\text{ L/min}$) and the target concentration is maintained at $C=3.0 vol.\%$, the governing equations simplify significantly.

In dynamic evaporation, the volumetric concentration is defined as:

$$C=\frac{V_{\text{vap}}}{Q_{\text{total}}}=\frac{V_{\text{vap}}}{V_{\text{vap}}+Q_{g}}$$

1. Fundamental equations for flow split ($C=3.0 vol.\%$)

For any target total flow rate ($Q_{\text{total}}$):

a) Vapor flow rate ($V_{\text{vap}}$):

$$V_{\text{vap}}=C\cdot Q_{\text{total}}=0.030\cdot Q_{\text{total}}$$

b) Air flow rate ($Q_{g}$):

$$Q_{g}=\left( 1-C \right)\cdot Q_{\text{total}}=0.970\cdot Q_{\text{total}}$$

c) Liquid flow rate ($q_{l}$):

To find the required liquid delivery rate from $V_{\text{vap}}$:

$$q_{l}\left( \text{mL/min} \right)=\frac{V_{\text{vap}}}{\left( \frac{\rho}{M}\cdot V_{m} \right)}$$

For isoflurane at ${20}^{\circ}\text{C}$ and $1\text{ atm}$ ($\rho=1.496\text{ g/mL}$, $M=184.49\text{ g/mol}$, $V_{m}=24.055\text{ L/mol}$):

$$\left( \frac{\rho}{M}\cdot V_{m} \right)=0.19505\text{ L/mL}$$

Substituting $V_{\text{vap}}=0.030\cdot Q_{\text{total}}$:

$$q_{l}\left( \text{mL/min} \right)=\frac{0.030\cdot Q_{\text{total}}}{0.19505}=0.1538\cdot Q_{\text{total}}$$

Converting to volumetric pump units ($\text{mL/h}$):

$$q_{l}\left( \text{mL/h} \right)=0.1538\cdot Q_{\text{total}}\times60=9.228\cdot Q_{\text{total}}$$

**2. Calculated Values for** $\boldsymbol{Q}_{\text{total}}\boldsymbol{=1.0,1.5,}\text{and }\boldsymbol{2.0}\text{ L/min}$

The equations applied across the three specific total flow conditions:

**Case 1: Total Flow Rate** $\boldsymbol{Q}_{\text{total}}\boldsymbol{=1.0}\text{ L/min}$

Vapor flow rate ($V_{\text{vap}}$): $0.030\times1.0\text{ L/min}=0.030\text{ L/min}\quad\left( 30\text{ mL/min} \right)$

Air flow rate ($Q_{g}$): $0.970\times1.0\text{ L/min}=0.970\text{ L/min}\quad\left( 970\text{ mL/min} \right)$

Liquid flow rate ($q_{l}$): $0.1538\times1.0=0.1538\text{ mL/min}\quad\left( 9.23\text{ mL/h} \right)$

**Case 2: Total Flow Rate** $\boldsymbol{Q}_{\text{total}}\boldsymbol{=1.5}\text{ L/min}$

Vapor flow rate ($V_{\text{vap}}$): $0.030\times1.5\text{ L/min}=0.045\text{ L/min}\quad\left( 45\text{ mL/min} \right)$

Air flow rate ($Q_{g}$): $0.970\times1.5\text{ L/min}=1.455\text{ L/min}\quad\left( 1455\text{ mL/min} \right)$

Liquid flow Rate ($q_{l}$): $0.1538\times1.5=0.2307\text{ mL/min}\quad\left( 13.84\text{ mL/h} \right)$

**Case 3: Total Flow Rate** $\boldsymbol{Q}_{\text{total}}\boldsymbol{=2.0}\text{ L/min}$

Vapor flow rate ($V_{\text{vap}}$): $0.030\times2.0\text{ L/min}=0.060\text{ L/min}\quad\left( 60\text{ mL/min} \right)$

Air flow rate ($Q_{g}$): $0.970\times2.0\text{ L/min}=1.940\text{ L/min}\quad\left( 1940\text{ mL/min} \right)$

Liquid flow rate ($q_{l}$): $0.1538\times2.0=0.3076\text{ mL/min}\quad\left( 18.46\text{ mL/h} \right)$

**Operational Note:** These values are subject to minor adjustments if the liquid pump only accepts integer values (does not support decimals).
